# Protein Interaction Networks by Proteome Peptide Scanning

**DOI:** 10.1371/journal.pbio.0020014

**Published:** 2004-01-20

**Authors:** Christiane Landgraf, Simona Panni, Luisa Montecchi-Palazzi, Luisa Castagnoli, Jens Schneider-Mergener, Rudolf Volkmer-Engert, Gianni Cesareni

**Affiliations:** **1**Institut für Medizinische Immunologie, Humboldt-Universität zu BerlinBerlinGermany; **2**Department of Biology, University of Rome “Tor Vergata,”RomeItaly; **3**Jerini AGBerlinGermany

## Abstract

A substantial proportion of protein interactions relies on small domains binding to short peptides in the partner proteins. Many of these interactions are relatively low affinity and transient, and they impact on signal transduction. However, neither the number of potential interactions mediated by each domain nor the degree of promiscuity at a whole proteome level has been investigated. We have used a combination of phage display and SPOT synthesis to discover all the peptides in the yeast proteome that have the potential to bind to eight SH3 domains. We first identified the peptides that match a relaxed consensus, as deduced from peptides selected by phage display experiments. Next, we synthesized all the matching peptides at high density on a cellulose membrane, and we probed them directly with the SH3 domains. The domains that we have studied were grouped by this approach into five classes with partially overlapping specificity. Within the classes, however, the domains display a high promiscuity and bind to a large number of common targets with comparable affinity. We estimate that the yeast proteome contains as few as six peptides that bind to the Abp1 SH3 domain with a dissociation constant lower than 100 μM, while it contains as many as 50–80 peptides with corresponding affinity for the SH3 domain of Yfr024c. All the targets of the Abp1 SH3 domain, identified by this approach, bind to the native protein in vivo, as shown by coimmunoprecipitation experiments. Finally, we demonstrate that this strategy can be extended to the analysis of the entire human proteome. We have developed an approach, named WISE (whole interactome scanning experiment), that permits rapid and reliable identification of the partners of any peptide recognition module by peptide scanning of a proteome. Since the SPOT synthesis approach is semiquantitative and provides an approximation of the dissociation constants of the several thousands of interactions that are simultaneously analyzed in an array format, the likelihood of each interaction occurring in any given physiological settings can be evaluated. WISE can be easily extended to a variety of protein interaction domains, including those binding to modified peptides, thereby offering a powerful proteomic tool to help completing a full description of the cell interactome.

## Abstract 2

By combining phage display and SPOT selection, the binding partners of any peptide recognition motif can be identified, thus facilitating the identification of all protein-protein interactions within a proteome

## Introduction

Protein–protein interactions govern cell physiology, and the disruption of some sensitive connections in the network can have pathological effects. Once a genome has been sequenced, one of the goals of functional genomics is the elucidation of the protein interaction network supporting biochemical and genetic pathways. Eventually, the aim is to study the consequences on cell physiology of disrupting the specific interaction between any two given proteins. Over the past few years, a number of high-throughput strategies have been proposed to achieve this goal (Uetz et al. 2000; Ito et al. 2001; Gavin et al. 2002; Ho et al. 2002). These endeavors demonstrated the feasibility of a proteomic approach to the protein interaction problem. However, the lack of a substantial overlap between the results of projects designed to cover the entire interactome of Saccharomyces cerevisiae emphasized the importance of confirming any interaction by different methods (von Mering et al. 2002).

An in vitro strategy that has received considerable attention is based on the production of proteins in a high-throughput fashion and on their analysis in an array format (Zhu and Snyder 2003). This approach is not limited to the study of protein interactions, and various other protein functions, including enzymatic activities, can be tested in the array format. However, although several experimental strategies are being explored, it is not yet clear which percentage of a eukaryotic proteome can be produced in a folded form in conventional expression systems and still remain functional once printed onto a solid support. High-density arrays of relatively short peptide chains, on the other hand, can be efficiently synthesized by a positionally addressable synthesis of peptides on cellulose membranes (SPOT synthesis) and have been used to facilitate mapping of antibody epitopes and more generally to study protein binding specificity (Frank 1992; Kramer and Schneider-Mergener 1998; Reineke et al. 2001).

The clear advantage of the array format could then be fully exploited to study protein interaction in those cases in which one of the partners participates in complex formation by docking a relatively short peptide into a receptor protein. In fact, a fairly large set of protein interactions are mediated by families of protein-binding domains (SH2, WW, SH3, PDZ, etc.) that act as receptors to accommodate, in their binding pockets, short peptides in an extended conformation (Pawson and Scott 1997; Pawson et al. 2002; Pawson and Nash 2003).

We have recently shown that the peptide sequences, obtained by panning phage-displayed random peptide libraries with SH3 domains, can be used to derive position-specific scoring matrices to computationally scan the entire proteome in search of putative partners (Tong et al. 2002). This approach is affected by relatively low accuracy and/or coverage, depending on the threshold score that is set in the predictive algorithm. As a consequence, reliable inferences are only achieved by considering the intersection of the network obtained by the phage display and the one obtained by a completely unrelated (orthogonal) technique, such as the yeast two-hybrid method (Tong et al. 2002).

**Figure 1:**
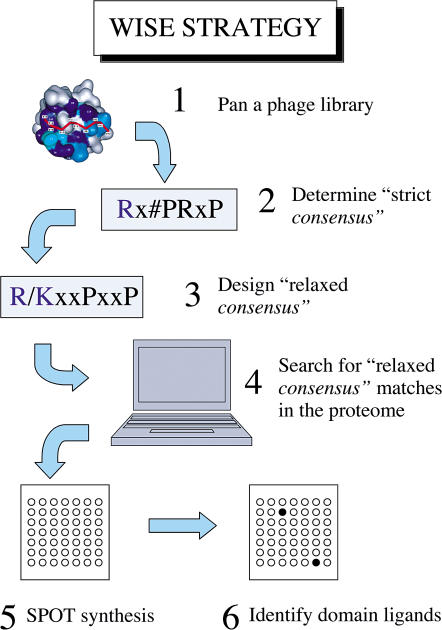
Schematic Representation of the WISE Strategy

We reasoned that an alternative strategy whereby the domain of interest is challenged with the entire collection of peptides that the domain is likely to encounter in the cell could eliminate one source of error. However, this straightforward approach is technically not feasible because the number of short peptides, even in a proteome as simple as the one of baker's yeast, is in the order of 10^7^. This figure is far beyond the limits of the current technology for peptide synthesis. On the other hand, one could use the information obtained from screening random peptide repertoires to filter out the amino acid sequences that are highly unlikely to bind, thereby decreasing the peptide sequence space to be tested experimentally. We will refer to this approach with the acronym WISE (whole interactome scanning experiment) (Figure 1).

It should be pointed out that WISE only addresses the problem of identifying natural peptides with the potential for binding to any given recognition domain. Although we use this information to infer the formation of protein complexes in vivo, there are a number of reasons why this inference could turn out to be incorrect. For instance, a peptide could be unavailable for interaction in the native protein structural context. Alternatively, the two inferred partners could be located in different cell compartments or expressed in different tissues or at different times during development. Finally, all the interactions that are mediated by an extended region of a protein surface and that cannot be supported by a relatively short peptide will be missed by this approach.

## Results

To assess the feasibility of this strategy, we have chosen eight S. cerevisaie proteins that contain SH3 domains belonging to five different specificity classes, as determined by phage display experiments (Tong et al. 2002). The SH3 domains of Rvs167 (P39743), Yfr024c (P43603), and Ysc84 (P32793) bind to peptides that conform to typical class 1 (RxxPxxP) and class 2 (PxxPxR) motifs. The SH3 domains of Boi1 (P38041) and Boi2 (P39969) bind to peptides that also match class 1 or class 2 motifs but that display a somewhat higher complexity and variability. By contrast, the SH3 domain of Sho1 (P40073) and Myo5 (Q04439) were found to bind only to class 1 peptides, with a preference either for positively charged or for large hydrophobic sidechains at position P-2, respectively. The SH3 binding motifs' residue nomenclature (P-0 being the first Pro in the PxxP motif) is according to Lim et al. (1994). Finally, the SH3 domain of Abp1 (P15891) was poorly defined by the phage display experiments, possibly because peptides longer than nine amino acids are required for efficient binding.

For each SH3 domain, we have defined a “relaxed pattern,” less selective than the pattern identified by comparing the most frequent ligands discovered by phage display. We have then used this to scan the whole S. cerevisiae proteome in search of peptides matching that pattern. The yeast proteome was searched with the program PatMatch at the *Saccharomyces* Genome Database (http://genome-www.stanford.edu/Saccharomyces/). A detailed description of the relaxed patterns can be found in Table S1. For instance, for class 1 peptides bound by the Rvs167 SH3 domain, instead of using the strict consensus motif RxFPxPP, we have defined a relaxed consensus by allowing either Arg or Lys at position P-3 and any amino acid at P-1 and P+2 (R/KxxPxxP). Standard conventions are used for representing consensus sequences of peptide ligands and protein modules (Aasland et al. 2002) and for the nomenclature of residue positions in SH3 ligands (Mayer 2001). We have to emphasize that this strategy is only suitable for identifying SH3 partners whose ligand domain can be confined to a short peptide detectable by the phage display approach. Although this is often the case, we need to realize that some SH3 domain interactions require more extended binding surfaces. As a consequence, they will not be identified by this approach (Barnett et al. 2000).

This approach was repeated for the eight SH3 domains. For each domain, approximately 1,500 peptides, matching the relaxed patterns, were selected for synthesis (see Datasets S1–S8). The peptides were synthesized at high density on cellulose membranes by SPOT synthesis technology, and the membranes were probed with the corresponding SH3 domain fused to glutathione S-transferase (GST). Finally, the bound domains were revealed by an anti-GST antibody and by a secondary anti-immunoglobulin G (IgG) antibody coupled to horseradish peroxidase (POD). The intensity of each SPOT was measured quantitatively in Boehringer light units (BLUs) (arbitrary light intensity units measured by a Lumi-Imager^ TM^ instrument)

**Figure 2:**
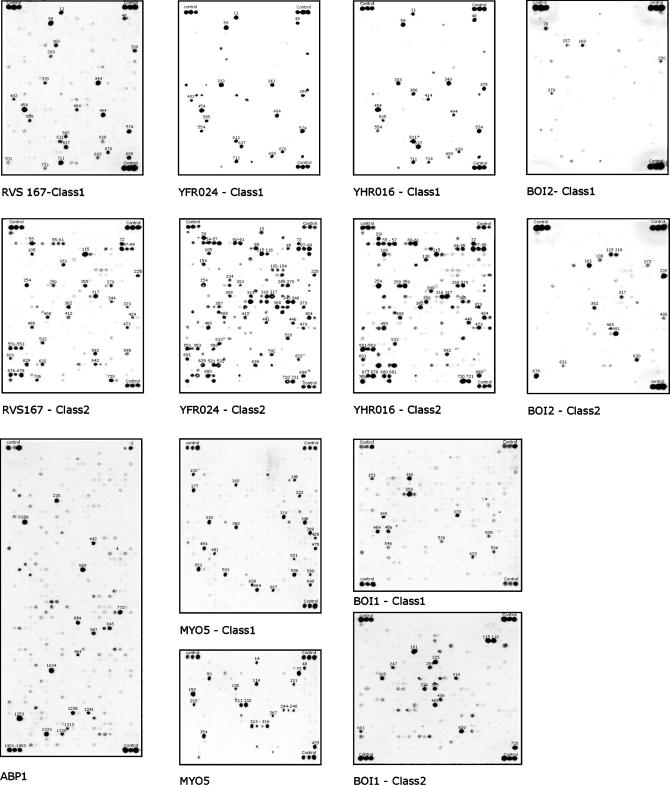
WISE Screening of the Binding Potential of Yeast SH3 Domains Seven GST–SH3 domain fusion proteins were challenged with peptides that match different relaxed consensi: class 1 (R/K)xxPxxP and class 2 PxxPx(R/K) . The Myo5 SH3 domain was also tested with peptides matching (F/P/L/W/A/E)xx(W/Y/L/M/F/H)xxPxxP, while the Abp1 membrane contains peptides matching either xxPx(K/R)P or Pxxx(K/R)P. In the design of these relaxed patterns, we first aimed at defining regular expressions that could retrieve from the proteome all the peptides that had been demonstrated, to bind to the domain under consideration. Whenever the number of matching peptides did not exceed an arbitrary chosen threshold of 1,500, we used subjective considerations about sidechain similarities to further relax the search pattern. The three spots near the membrane corners contain peptides that bind to the anti-GST antibody. The intensity of these spots was used for normalization.

**Figure 3:**
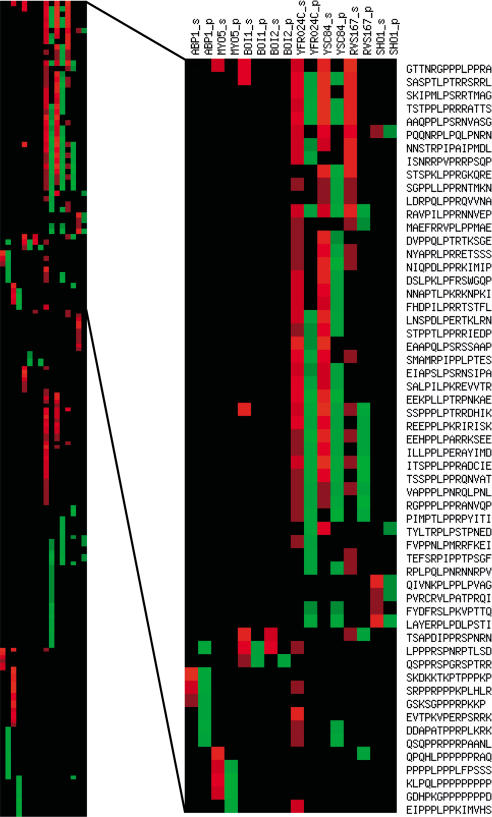
Comparison of the Phage Display Prediction and the Results of the SPOT Binding Test by the WISE Approach The quantitative results of the experiments in Figure 1 are visualized with a graphical representation obtained with the tool EPCLUST available at http://ep.ebi.ac.uk/EP/EPCLUST. The PepSpot data, represented in red in a semiquantitative scale, is compared to the phage display prediction. Only peptides with BLUs (measured on a Lumi-Imager^TM^) higher than 25K are included in the representation. The red intensity scale corresponds to BLU values in the ranges 25K–35K, 35K–45K, 45K–55K, 55K–85K, and larger than 85K, where higher BLU values correspond to a brighter red. Peptides that obtained a high score with the phage display-derived position-specific scoring matrix (Tong et al. 2002) are in brighter green. Peptides with a lower score are represented with a correspondingly lighter green according to an arbitrary linear scale.

Figure 2 and Datasets S1–S8 report the results of these experiments in which the pattern of the reactive spot forms a sort of fingerprint that defines the recognition specificity of the specific SH3 domain. The differences and similarities in recognition specificity are better appreciated in the representation of Figure 3, where the red hue of the small horizontal bars indicate the intensity of the binding reaction of a specific peptide for each SH3 domain column. As expected from the phage display experiment, the SH3 domains of Rvs167, Yfr024c, and Ysc84 have overlapping specificities, with Rvs167 proving more selective and Yfr024c more promiscuous. By contrast, the peptides that bind to the Abp1 (P15891) and Myo5 SH3 domains are characterized by different motifs. The results reported in Figure 3 also point out that peptide recognition patterns inferred from the phage display experiments (green in Figure 3) overlap only partially with the SPOT recognition patterns. These differences are particularly apparent in the case of Boi1 and Boi2. For these domains, the data obtained by phage display have proven insufficient for the target peptides to be inferred with sufficient accuracy, since only three out of 15 peptides have been predicted correctly. This comparison underlines the danger of using regular expressions or position-specific scoring matrices, derived from a relatively small number of peptide sequences, for inferring new peptide targets.

### Correlation of the SPOT Quantitative Output and Dissociation Constant

**Figure 4:**
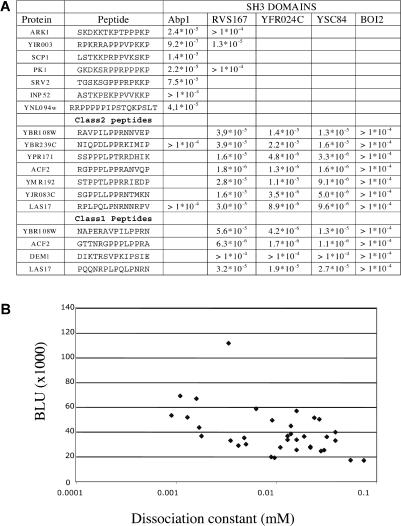
Measurement of Dissociation Constants and Correlation with SPOT Intensities (A) Dissociation constants were measured with a BIAcoreX instrument as described in the Materials and Methods. The experiments with the Abp1 SH3 domain were carried out in triplicate. (B) Normalized BLU intensities plotted as a function of the log of the dissociation constant.

**Figure 5:**
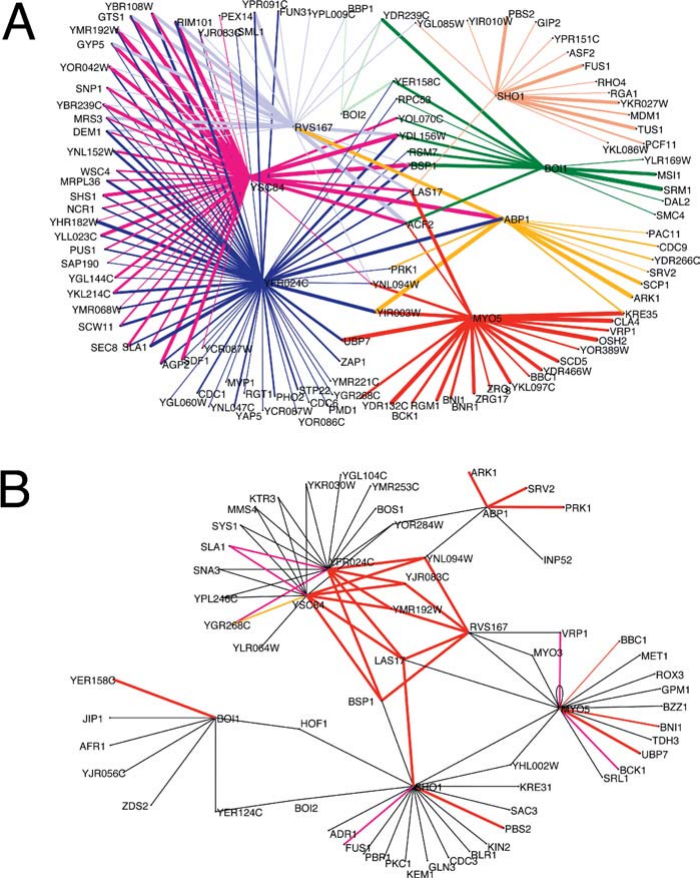
Inferred Protein Interaction Networks (A) Protein interaction network mediated by the SH3 domains of the proteins characterized in this study. The SH3-containing proteins are represented as blue dots, while the prey partner proteins are represented as black dots. The interactions mediated by each SH3 are represented in a different color, and the edge thicknesses are proportional to the BLU intensity of the corresponding interaction, according to the scale described in Figure 3. (B) The graph represents the interaction network mediated by the SH3 domains of Rvs167, Ysc84, Yfr024c, Abp1, Myo5, Sho1, Boi1, and Boi2 as determined by the two-hybrid approach (Tong et al. 2002). The interactions (edges) that were confirmed by our WISE method (BLU value higher than 25K) are colored in red or magenta. The interactions in magenta, differently from the ones in red, were not correctly inferred by the phage display approach. The interaction in orange was inferred by the phage display approach, but not confirmed by the WISE method. The network was visualized by the Pajek package (http://vlado.fmf.uni-lj.si/pub/networks/pajek/).

The sensitivity of the SPOT interaction experiment is such that even peptides with a dissociation constant as high as 10^−4^ M or above give a positive signal in the assay (Kramer et al. 1999). To establish a correlation between affinity and the BLU signal, we have measured, by surface plasmon resonance, the dissociation constants of a number of peptides that were positive in the membrane assay (Figure 4A). The dissociation constants ranged from 9.4 × 10^−7^ M to values that, being larger than 10^−4^ M, could not be confidently measured. As previously observed for antibodies (Kramer et al. 1999), in these experiments signal intensity also correlated inversely with the dissociation constant (correlation coefficient of –0.4; Figure 4B). This correlation was obtained by comparing experiments performed with different probes and different membranes and can be further improved through more careful standardization (C. Landgraf and R. Volkmer-Engert, unpublished data). Thus, this approach, in contrast with other high-throughput approaches, is accompanied by a quantitative output that correlates, albeit partially, with the dissociation constant. As such, it can be used to assign figures to the edges of the inferred interaction network. This is illustrated in Figure 5A, where the inferred SH3-mediated interaction network is represented by different colors to differentiate interaction mediated by different SH3 domains and different edge thicknesses in order to distinguish interactions with different affinities.

### Inferred Protein Ligands Share Common Functions

Interacting proteins often share similar functions and participate in common processes. Hence, we examined whether the proteins, found in our approach to bind to a specific SH3 domain, could be preferentially associated to a biological process. For this analysis we considered, as putative ligands, all the proteins containing at least one peptide with an intensity higher than an arbitrarily chosen threshold of 20,000 (in BLUs, corresponding to a dissociation constant of approximately 100 μM). We then used the FunSpec software (Robinson et al. 2002) to identify the Gene Ontology (GO) terms significantly enriched in the list of proteins interacting with any specific SH3 domains. FunSpec uses a hypergeometric distribution to evaluate the probability (*p*) that the intersection of a protein list with any given functional category occurs by chance. The inferred ligands of the Ysc84, Yfr024c, and Rvs167 SH3 domains were significantly enriched for the GO biological process term “actin cytoskeleton organization and biogenesis” (*p* < 5.46 × 10^−7^, *p* < 7.06 × 10^−6^, and *p* < 5.50 × 10^−5^, respectively). By contrast, the partners of Abp1 and Myo5 SH3 domains were found to be enriched for the GO terms “actin cortical patch assembly” (*p* < 3.49 × 10^−7^) and “actin cytoskeleton” (*p* < 7.14 × 10^−6^). These results are in accord with the available information about the participation of these bait proteins in the organization of the yeast cytoskeleton, whereas arbitrarily selected gene groups of similar size showed no comparable enrichments for any of the GO terms (best result, *p* < 10^−3^).

We have previously shown that the information obtained by panning random peptide libraries can be used to draw an interaction network that recapitulates a fraction of the SH3-mediated interaction network determined by the two-hybrid approach (Tong et al. 2002). By using inferred networks of comparable size, the intersection with the two-hybrid network was larger for the WISE than for the phage display network, including three more proteins and six new edges (Figure 5B). Furthermore, as shown below, at least some of the WISE interactions, not present in the two-hybrid network, can be shown to occur in physiological conditions in yeast.

### The Tightest SH3 Peptide Ligands Mediate Complex Formation In Vivo

The Abp1 SH3 domain, compared to most of the remaining SH3 domains that we have studied, has a narrower peptide recognition specificity and, as a consequence, fewer inferred partners. Our analysis confirmed that Srv2 and Ark1, previously identified Abp1 SH3 functional partners (Lila and Drubin 1997; Fazi et al. 2002), contain peptides that bind with an affinity in the 1–100 μM range. Furthermore, fragments of Prk1 (P40494), Yir003w (P40563), and Ynl094w (P53933) were reported to bind to the Abp1 SH3 domain in vitro (Fazi et al. 2002). Surprisingly, we could not identify any tetradecapeptide in the Ynl094w protein with affinity better than 100 μM. We noticed, though, that if we extend the peptide RRPPPPPIPSTQKP (predicted to be a ligand of the Abp1 SH3 domain by a variety of approaches) to include three more residues at the C-terminus, the affinity rises to approximately 40 μM. Finally, we identified Scp1 (Q08873), the yeast homolog of calponin, as a putative new Abp1 partner.

**Figure 6:**
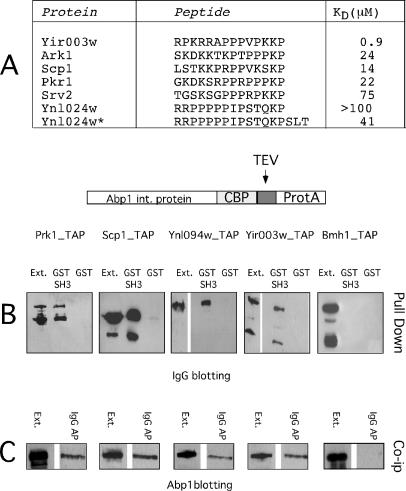
Characterization of Abp1 Ligands (A) The dissociation constants of the 11 peptides that bound most efficiently to the Abp1 SH3 domain in the SPOT synthesis assay were measured by BIAcore experiments. (See also Table S1.) The results of the experiments for the peptides with the highest affinity are reported here. (B) The genes encoding the putative Abp1 ligands (Prk1, Yir003w, Scp1, and Ynl094w) were modified by the TAP technology to produce tagged proteins. A strain expressing the “tapped” Bmh1 protein is used as a control. Yeast extracts encoding the tagged proteins were used in pulldown experiments in the presence of 100 μg of GST–Abp1 SH3 or GST alone as a negative control. The “Ext.” lane was loaded with 1/20 of the extract used in the pulldown experiment. (C) The same extracts were affinity-purified on an IgG affinity resin and then the affinity tag, protein A, released by cutting with the TEV protease. The proteins that were copurified with the “tapped” baits were revealed with an anti-Abp1 serum.

In order to assess how accurately peptide binding affinity permits us to infer physiological partners, we investigated whether the putative partners can be copurified with Abp1 in vivo. We used the tandem affinity purification (TAP) technology (Rigaut et al. 1999) to tag the Prk1, Ynl094w, Scp1, and Yir003w proteins, and we initially asked, by pulldown assays, whether the putative peptide targets of the Abp1 SH3 domain were available for interaction in the protein natural context. As seen in Figure 6A, the four proteins identified by our approach can be affinity-purified on a sepharose resin containing the Abp1 SH3 domain, thus indicating that the target peptides can bind in their native protein context.

To establish whether Abp1 forms a complex with these proteins in vivo, we next affinity-purified the four tagged proteins on an IgG resin. We next probed the purified complexes with an anti-Abp1 antibody. As shown in Figure 6B, Abp1 could be copurified with Prk1, Scp1, Ynl094w, and Yir003w, but not with Bmh1, used as a negative control. In conclusion, at least in the case of Abp1, the search for the tightest binding peptides in the whole yeast proteome led to the to identification of proteins that form a complex with the bait domain when expressed at physiological levels in vivo.

We have also investigated whether the fraction of coimmunoprecpitated Abp1 protein correlates with either the BLU intensity or the dissociation constant of the corresponding SH3–peptide interaction. The observed lack of correlation indicates that other factors, as, for instance, local protein concentration (mediated by different interactions), are important in determining the efficiency of complex formation.

### WISE Scanning of the Human Proteome

**Figure 7:**
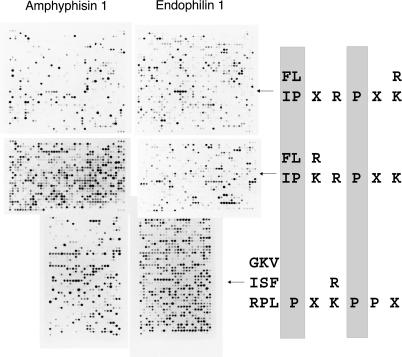
Scanning of the Human Proteome in Search of Ligands for the Amphiphysin and Endophilin SH3 Domains The relaxed target peptide consensi (right) were derived from the available phage display experimental data and used to search the human proteins contained in the SwissProt/TREMBL database with the software ScanProsite, found at http://us.expasy.org/tools/scanprosite/.

Finally, we asked whether this approach could be extended to the analysis of a mammalian proteome that is approximately five to six times more complex than the yeast one. To this end, we selected two proteins involved in membrane recycling, amphiphysin-1 (P49418) and endophilin-1 (Q99962), and whose SH3 domains we had previously characterized by phage display (Cestra et al. 1999). These two SH3 domains are also known to have overlapping recognition specificity, although their preferred target peptides are different and the overall recognition specificity differs from the ones of the yeast SH3 domains characterized so far by this approach (see Figure 2). We have screened with the amphiphysin and endophilin SH3 domains all the peptides in the SwissProt/TREMBL database that contain the (P/F/l/I)XRPXX(R/K), the (P/F/l/I)(K/R)RP, or the (P/l/R/F/S/I/V/K/G)PX(R/K)PP motifs. Because of the redundancy of the SwissProt/TREMBL database and because the peptide families matching the three motifs overlap, some of the total 3,774 peptides were synthesized several times, thereby providing an internal control of the approach's reproducibility (Figure 7; Datasets S9 and S10).

Dynamin and synaptojanin, two proteins involved in endocytosis, form an SH3-mediated complex with amphiphysin and endophilin in vivo (McPherson et al. 1996; de Heuvel et al. 1997; Ringstad et al. 1997). Our approach identified in both proteins at least one peptide that is a ligand for the amphiphysin and endophilin SH3 domains. Interestingly, other proteins that have been already implicated in endocytosis and its control (but not yet described as physiological partners of amphiphysin and endophilin) contain peptides that are ranked among the highest affinity ligands by our approach. Several other proteins of unknown function are predicted to bind to the SH3 domain of these two proteins.

## Discussion

The WISE strategy described here has the merit of combining the strengths of a selective approach (such as panning combinatorial peptide libraries displayed on phage) with a quantitative analysis that can be achieved by screening a large number of peptides arrayed at high density on a solid support. This makes it possible to identify rapidly and directly the tighest ligands of a peptide-binding receptor among all the peptides in an entire proteome. We have demonstrated the approach by applying it to the family of SH3 domains. However, WISE can also be extended to all those domain families (WW, PDZ, EH, GYF, VHS, SH2 PTB, 14-3-3, FHA, WD40, etc.) that mostly recognize short peptides in their partner proteins.

Our approach, as any in vitro approach, suffers from some simplifications when it comes to inferring the physiological partners from the domain–peptide interaction data. According to a naive strategy, we would assimilate the cell to a cellulose membrane, where all the peptides are equally represented and accessible to the bait domain, and we would be tempted to conclude that all the proteins containing the identified peptide ligands were likely to be physiological partners. In the real cell, however, the target peptides may be hidden inside the core of the folded proteins, and the protein partners may not be equally represented. Furthermore, the partner proteins may be expressed in different cell types or segregated in different macromolecular complexes or cell compartments. In order to obtain more reliable inferences, the peptide interaction information obtained by a WISE approach should be complemented by information about peptide accessibility obtained by structural predictors (Garner et al. 1998; Linding et al. 2003) and data about mRNA and protein concentrations in different physiological and subcellular contexts (Simpson et al. 2000; Kumar et al. 2002).

Nevertheless, the average number of peptides in the yeast proteome that have the potential to bind SH3 domains with an affinity that may have physiological relevance was found to be surprisingly high, ranging from a few peptides, in the case of the Abp1 and Boi2 SH3 domains, to several tenths, in the case, for instance, of the Yfr024w SH3 domain. Given the hypothesis that all (or most of) these peptides are equally expressed inside the cell and exposed to the solvent in the folded protein structure as most Pro-rich peptides are, these findings raise the question of whether the observed binding promiscuity has any physiological implication. Recent proteome-wide analyses of yeast protein complexes have revealed that many proteins are organized in discrete complexes (Gavin et al. 2002; Ho et al. 2002). Yet this approach has failed to identify a large number of interactions whose physiological relevance was validated by traditional single (or few) protein studies, implying that many physiologically relevant protein interactions do not lead to the formation of stable complexes. SH3-mediated interactions may belong to this latter class. This is consistent with the observation that SH3-containing proteins have a connectivity significantly lower than average (2.33; average, 4.00) in the yeast complexosome (Gavin et al. 2002; Ho et al. 2002), in contrast with the observed connectivity in the interaction network derived from high-throughput two-hybrid experiments (average connectivity of SH3-containing proteins, 5.05; average connectivity for all proteins, 1.53) (Uetz et al. 2000; Ito et al. 2001; L. Montecchi-Palazzi and G. Cesareni, unpublished data). SH3-mediated interactions are much less likely to be detected by coimmunoprecipitation assays than by solid-state (or two-hybrid) assays, because relatively weak interactions are almost certainly lost in the extensive washing needed for coimmunoprecipitation experiments. Our approach has made it possible to rediscover most of the SH3-mediated protein interactions that were previously described for these proteins. Admittedly, though, few clearly characterized protein interactions of this type have yet been reported in the literature. The few failures of our approach (false negatives) are due to weaknesses in the design of the relaxed consensus used to search for matching peptides in the protein databases.

All the same, we have identified a larger number of target peptides that bind with affinities that are comparable to the ones of the validated physiological targets. Some of these peptides may never encounter the cognate SH3 domain, while some will only meet partners in specific physiological conditions. Others may contribute to add specificity to the formation of a complex by cooperating with other associated low-specificity binding domains. Finally, we have to consider a new scenario in which proteins, even when not forming stable complexes, are seldom isolated in solution, but navigate in the cell by moving from one weak partner to another. These weak interactions may be important in modulating cell architecture even when they are not instrumental in the nucleation of a stable complex. Although this is difficult to prove, the semiquantitative data provided by our approach, complemented with the results of large-scale expression and localization studies, may eventually allow one to model these different settings.

The in vitro approach that we have described, albeit limited to interactions in which one of the partners can be reduced to a relatively short peptide, presents a number of interesting features that complement other strategies aimed at revealing the details of the protein interaction network within cells.

First, it takes full advantage both of the genomic information that is being accumulated and of the array format in which all the possible targets are equally represented. Second, it is comprehensive and provides a high level of detail on the interaction topology. Third, it is not affected by protein concentrations inside the cell and is very sensitive (interactions up to 100 μM can be detected). Fourth, interactions that depend on peptide modifications, for instance, phosphorylation, can also be studied. Fifth, the output is semiquantitative. Finally, the identified target peptide can be used as a lead to develop tighter binding molecules in order to interfere with complex formation in vivo.

We have shown that the current implementation of the SPOT synthesis technology is sufficient to carry out WISE screening of a proteome as complex as the one of a mammalian organism. Foreseeable technological improvements of the SPOT synthesis technology will permit the assembly of relatively cheap microarrays containing up to 15,000 peptides. This will extend the approach's power by relieving, in some cases, the requirement for an experimental filtering step, as performed here by the phage display approach, thereby allowing more freedom in the design of the relaxed pattern.

## Materials and Methods

### 

#### Genome tagging

Yeast PJ694a strains expressing TAP-tagged ORFs were constructed as described (Rigaut et al. 1999). Primers bearing a sequence identical to the C-terminal part of the ORF were used to amplify the TAP cassette. Primers for Yir003 are forward: GACGTTGATTCTGCCTTACATTCAGAAGAAGCGTCTTTTCACTCCCTTTCCATGGAAAAGAGAAGATG and reverse: CCATTATTATTAATAACACCTCTAGTTTGCTCGTCATTCACATATTTCTACGACTCACTATAGGGCGA. Primers for Scp1 are forward: TCTCAGGCTACTGAAGGAGTGGTGTTAGGACAACGGAGAGATATAGTTCCATGGAAAAGAGAAGATG and reverse: GGAAAACTAAAATATATCAAAGGAACTTTGGTTGCGTATATAGGGTTCTACGACTCACTATAGGGCGA. Primers for Prk1 are forward: GTAGATGATTTAGAAGCCGATTTTAGAAAAAGGTTTCCCAGCAAAGTTTCCATGGAAAAGAGAAGATG and reverse: AAAAATTTCAAATGATTGACGAAAGAAAATTTGTACATTTTGTATGACTACGACTCACTATAGGGCGA. Primers for Ynl094w are forward: TTAAGTTTGGAAGACAGTATTCGCAGAATTAGGGAGAAGTATTCAAACTCCATGGAAAAGAGAAGATG and reverse: CACTCTAAAACGTTGAAAATGGCTCCAATTCATAAGGTCACTTTAGTGTACGACTCACTATAGGGCGA.

The polymerase chain reaction (PCR) fragments were used to transform the yeast strain. Positive clones were selected on selective plates and checked by PCR analysis and Western blot analysis. For the PCR analysis, we used a new forward primer together with the reverse ones used for the construction: forward for Yir003, AGCAGATGGAGGACCAAATGGAGGTTG; forward for Scp1, CGGTTATATGAAAGGTGCATCTCAGGC; forward for Prk1, CGTTTACAATCAAAGAAACTGCCGATTG; and forward for Ynl094w, GGACTCAATTCAAAAATTGAGCAATCAAG.

#### Pulldown assay

Yeast strains expressing TAP-tagged Yir003w, Scp1, Prk1, Ynl094c, or Bmh1 as a control, were cultured at 30°C in 5-l flasks containing 2 l of YPD medium, collected in the exponential growth phase, and lysed mechanically with glass beads in 5 ml of IPP-150 buffer (10 mM Tris–HCl [pH 8.0], 150 mM NaCl, 0.1% NP-40) in the presence of protease inhibitors (2 mM benzamidine, 0.5 mM PMSF, 1 mM leupeptina, 2.6 mM aprotinin). Half of the extract was incubated for 2 h at 4°C with 100 μg of GST–Abp1SH3 (bound to glutathione–sepharose), while the remaining half was incubated with 100 μg of GST as a control. The resins were washed four times with 5 ml of IPP-150 buffer and the bound proteins recovered (by boiling in SDS–BLU–dye) and analyzed on a 10% SDS–polyacrylamide gel. They were transferred onto nitrocellulose membranes. Filters were blocked overnight at 4°C in PBS containing 5% milk powder (blocking solution), and then incubated with peroxidase (POD)-conjugated anti-POD antibody (PAP) antibody (Sigma P-2026; Sigma, St. Louis, Missouri, United States) diluted 1:1,000 for 2 h at room temperature (RT), washed five times for 15 min with PBS–0.05% Tween, and revealed by chemoluminescence. GST fusion proteins were expressed and purified by standard procedures.

#### Coimmunoprecipitation

Yeast cultures expressing TAP-tagged Yir003, Scp1, Prk1, Ynl094c, or Bmh1 were cultured, collected, and lysed as described for the pulldown experiments. Each extract was incubated with 500 μl of IgG–sepharose (Pharmacia Biotech 17–0969-01; Amersham Pharmacia, Uppsala, Sweden) for 2 h at 4°C. The resins were washed four times in 5 ml of IPP-150 buffer, resuspended in 300 ml of 50 mM Tris–HCl (pH 8), 0.5 mM EDTA, 5 mM DTT, transferred to Eppendorf tubes, and incubated with 30 U of recombinant TEV protease (Invitrogen 10127–017; Invitrogen, Carlsbad, California, United States) for 1 h at 20°C. After centrifugation for 2 min at 2,300 rpm, the supernatants were loaded on 10% SDS–polyacrylamide gels and then transferred onto nitrocellulose membranes. Filters were blocked overnight at 4°C in blocking solution, incubated for 2 h at RT with anti-Abp1 antibody (diluited 1:1,000), and washed five times with PBS–0.05% Tween. They were then incubated for 1 h at RT with anti-rabbit POD coniugated, washed ten times with PBS–0.05% Tween, and detected by chemoluminescence.

#### Peptide array synthesis

Cellulose membrane-bound peptides were automatically prepared according to standard SPOT synthesis protocols (Frank 1992) using a Spot synthesizer (Abimed, Langenfeld, Germany) as described in detail (Kramer and Schneider-Mergener 1998). For generation of the sequence files, the software LISA (Jerini AG, Berlin, Germany) was used. To exclude false-positive spots in the incubation experiment, all Cys were replaced by Ser. The generated arrays of 15mer peptides were synthesized on cellulose-(3-amino-2-hydroxy-propyl)-ether (CAPE) membranes, because of a better signal-to-noise ratio in the incubation experiments.

#### Preparation of CAPE membranes

A 18 × 28 cm Whatman 50 paper (Whatman, Maidstone, United Kingdom) was immersed in a stainless steel dish containing a solution of 400 mg of *p*-toluenesulfonic acid in methanol (50 ml) and shaken for 3 min. The membrane was removed from the tray and air-dried. Meanwhile a solution of 7.8 g of N-(2,3-epoxypropyl)-phathalimid in dioxane (60 ml) was heated up to 80°C in a covered stainless steel dish placed on a shaking platform using a heater mat. Then, a solution of 400 mg of *p*-toluenesulfonic acid in 5 ml of dioxane was added. Immediately, the membrane was placed in this solution and shaken at 80°C for 3–5 h. Afterwards, the membrane was washed three times with 50 ml of dioxane and ethanol (twice, 50 ml each) and subsequently incubated with a 10% (v/v) solution (50 ml) of hydrazine hydrate (80%) in ethanol for approximately 6 h. Finally, the membrane was washed twice with ethanol, three times with dimethylacetamide, and once again with ethanol (twice, 50 ml each), and dried. The loading of this type of amino-functionalized cellulose membrane is about 120–200 nmol/cm^2^.

#### SH3 domain binding studies of cellulose-bound peptides

Generally, all incubations and washing steps were carried out under gentle shaking. After washing the membrane once with ethanol (10 min) and three times for 10 min with Tris-buffered saline (TBS: 50 mM Tris-(hydroxymethyl)-aminomethane, 137 mM NaCl, 2.7 mM KCl, adjusted to pH 8 with HCl), the membrane-bound peptide arrays were blocked (3 h) with blocking buffer. Blocking reagent (CRB, Northwich, United Kingdom) was diluted 1:10 in TBS containing 5% (w/v) sucrose. After washing with TBS (10 min), 10 μg/ml of the corresponding GST-fused SH3 domain (in blocking buffer) was added and incubated overnight at 4°C. After washing three times for 10 min with TBS, the anti-GST monoclonal antibody (mAb) (G1160; Sigma) was added at a concentration of 1 μg/ml in blocking buffer for 2 h at RT. Subsequently, the membrane was washed three times with TBS (10 min each) and the POD-labeled anti-mouse mAb (1 μg/ml in blocking buffer) was applied for 1.5 h at RT, followed by washing three times with TBS. Analysis and quantification of peptide-bound SH3 domains were carried out using a chemoluminescence substrate and the Lumi-Imager^TM^ instrument (Roche Diagnostics, Basel, Switzerland). For quantification, the SPOT signal intensities were measured in BLUs. To exclude false-positive results, in the SH3-incubation experiment, each membrane was preexamined with GST/anti-GST mAb/anti-mouse mAb. The data obtained with the different membranes were normalized by using as a reference the intensity of three control peptides that bind to the anti-GST antibody. The sequence of these peptides were QRALAKDLIVPRRP, LAKDLIVPRRPEWN, and DLVIRPPRPPKVLGL.

#### BIAcore analysis

Surface plasmon resonance measurements were carried out with a BIAcoreX instrument (BIAcore, Uppsala, Sweden). Experiments were carried out on sensor chips with GST-fused SH3 domains and GST as a control. GST-fused SH3 domains and the GST were coupled to CM5 sensor chips using the EDC/NHS (N-(3-dimethylaminopropyl)-N′-ethylcarbodiimide and N-hydroxysuccinimide)amine-coupling kit, yielding approximately 4,350 resonance units in the case of the GST-fused SH3 domain and 4,330 resonance units for GST. Interaction analysis was performed at 25°C with the peptides dissolved in 10 mM HEPES, 150 mM NaCl, 3.4 mM EDTA, and 0.005% surfactant P20 (pH 7.4), at 15 μl/min flow rate, using six to seven dilutions, ranging from 500 μM to 65 nM. Dissociation constant values were evaluated by applying the steady-state model using BIAcore evalution 3.1 software.

## Supporting Information

Dataset S1 Results of the SPOT Analysis Experiments for the Abp1 SH3 Domain (102 KB TXT).

Dataset S2 Results of the SPOT Analysis Experiments for the Boi1 SH3 Domain (104 KB TXT).

Dataset S3 Results of the SPOT Analysis Experiments for the Boi2 SH3 Domain (103 KB TXT).

Dataset S4 Results of the SPOT Analysis Experiments for the Myo5 SH3 Domain (86 KB TXT).

Dataset S5 Results of the SPOT Analysis Experiments for the Rvs167 SH3 Domain (104 KB TXT).

Dataset S6 Results of the SPOT Analysis Experiments for the Yfr024c SH3 Domain (104 KB TXT).

Dataset S7 Results of the SPOT Analysis Experiments for the Yhr016c SH3 Domain (104 KB TXT).

Dataset S8 Results of the SPOT Analysis Experiments for the Sho1 SH3 Domain (76 KB TXT).

Dataset S9 Results of the SPOT Analysis Experiments for the Amphyphisin SH3 Domain (183 KB TXT).

Dataset S10 Results of the SPOT Analysis Experiments for the Endophilin SH3 Domain (184 KB TXT).

Table S1 Design of Relaxed Consensi (66 KB PDF).

## Abbreviations

BLU: Boehringer light unit
CAPE: cellulose-(3-amino-2-hydroxy-propyl)-ether
EDC: N-(3-dimethylaminopropyl)-N′-ethylcarbodiimide
GO: Gene Ontology
GST: glutathione S-transferase
HNS: N-hydroxysuccinimide
IgG: immunoglobulin G
mAb: monoclonal antibody
PAP: peroxidase-conjugated anti-peroxidase antibody
PCR: polymerase chain reaction
POD: peroxidase
RT: room temperature
SPOT synthesis: synthesis of peptides on cellulose membranes
TAP: tandem affinity purification
TBS: Tris-buffered saline
WISE: whole interactome scanning experiment
