# Supplementary material for: Protein Interaction Networks by Proteome Peptide Scanning
Source: PLoS Biol. 2004 Jan 20;2(1):e14. doi: 10.1371/journal.pbio.0020014 (PMC314469; doi:10.1371/journal.pbio.0020014)
Supplement: Table S1 — (66 KB PDF). [file pbio.0020014.st001.pdf]

Table 1. Design of relaxed consensi .

	Phage display Consensus	Relaxed consensus	N of peptides screened
Rvs167	RxFPxPP<br>PP□PPR	(R/K)xxPxxP<br>PxxPx(R/K)	735<br>729
Yfr024c	PxLPxRP	(R/K)xxPxxP<br>PxxPx(R/K)	735<br>729
Ysc84	PxLPxR	(R/K)xxPxxP<br>PxxPx(R/K)	735<br>729
Boi1	RxxPxxP<br>pPRxPrR□	(R/K)xxPxxP<br>PxxPx(R/K)	735<br>729
Boi2	ppRnPxR□	(R/K)xxPxxP<br>PxxPx(R/K)	735<br>729
Abp1	+P□xxPx+P<br>PxxPxRP	xxPx(K/R)P<br>Pxxx(K/R)P	687<br>714
Myo5	Px@pPPxxP	(R/K)xxPxxP<br>(F/P/L/W/A/E)xx(W/Y/L/M/F/H)x<br>xPxxP	735<br>470
Amph1	PxRPxR	(P/F/L/I)(K/R)RP<br>(P/F/L/I)xRPx(R/K)<br>(P/L/R/F/S/I/V/K/G)Px(R/K)PP	1684<br>1139<br>951
End1	P+RPPxP	(P/F/L/I)(K/R)RP<br>(P/F/L/I)xRPx(R/K)<br>(P/L/R/F/S/I/V/K/G)Px(R/K)PP	1684<br>1139<br>951

@: aromatic residues; □ : hydrophobic residues; + : arginine or lysine; x: any residue.

The phage display *consensus* sequences were obtained by Tong et al (2002) and by Cestra et al (2001).
